# Mitochondrial SOS: how mtDNA may act as a stress signal in Alzheimer’s disease

**DOI:** 10.1186/s13195-023-01322-6

**Published:** 2023-10-11

**Authors:** Isabelle K. Gorham, Robert C. Barber, Harlan P. Jones, Nicole R. Phillips

**Affiliations:** 1 Department of Microbiology, Immunology, and Genetics, School of Biomedical Sciences, University of North Texas Health Science Center, Fort Worth, TX USA; 2 Department of Family Medicine, Texas College of Osteopathic Medicine, University of North Texas Health Science Center, Fort Worth, TX USA

**Keywords:** Mitochondria, Alzheimer’s Disease, Oxidative Stress, mtDNA, Inflammation

## Abstract

**Background:**

Alterations in mitochondrial DNA (mtDNA) levels have been observed in Alzheimer’s disease and are an area of research that shows promise as a useful biomarker. It is well known that not only are the mitochondria a key player in producing energy for the cell, but they also are known to interact in other important intracellular processes as well as extracellular signaling and communication.

**Body:**

This mini review explores how cells use mtDNA as a stress signal, particularly in Alzheimer’s disease. We investigate the measurement of these mtDNA alterations, the mechanisms of mtDNA release, and the immunological effects from the release of these stress signals.

**Conclusion:**

Literature indicates a correlation between the release of mtDNA in Alzheimer’s disease and increased immune responses, showing promise as a potential biomarker. However, several questions remain unanswered and there is great potential for future studies in this area.

Alzheimer’s disease (AD) is a neurodegenerative disease characterized by the accumulation of amyloid beta plaques, neurofibrillary tau tangles, and symptoms of cognitive impairment [1]. Despite a great deal of study, the pathophysiology of Alzheimer’s disease progression remains largely unclear. Although not used yet as a clinical biomarker [2], mitochondrial dysfunction and levels of mitochondrial DNA (mtDNA) detectable in blood or cerebrospinal fluid (CSF) are an area of AD research that offer potential in understanding the initiation and progression of AD.

## mtDNA in AD

AD pathology is known to begin prior to the emergence of clinical symptoms and initiation is thought to begin earlier than amyloid beta or tau is detectable [3]. There is evidence that alterations in mtDNA may be detectable in this early initiation period of AD that may be related to cognitive decline. Individuals with AD have been shown to have decreased levels of mtDNA in brain tissue when compared to cognitively normal individuals [4–6], and this decline in mtDNA present in the brain is also reflected in patient CSF [3, 7]. This loss of mtDNA was detectable in those with inherited and sporadic AD and was detectable up to a decade before the predicted onset of clinical AD symptoms [3]. Alterations of mtDNA levels have also been detectable in the blood; studies have shown decreased mtDNA present in the cellular fraction of blood [8–11] and increased levels of mtDNA present in the cell-free plasma fraction of the blood associated with both AD and regular aging [8, 12, 13]. This indicates that cellular mtDNA may be released extracellularly at an increased rate in those with AD.

## Mechanisms of mtDNA release

**Fig. 1:**
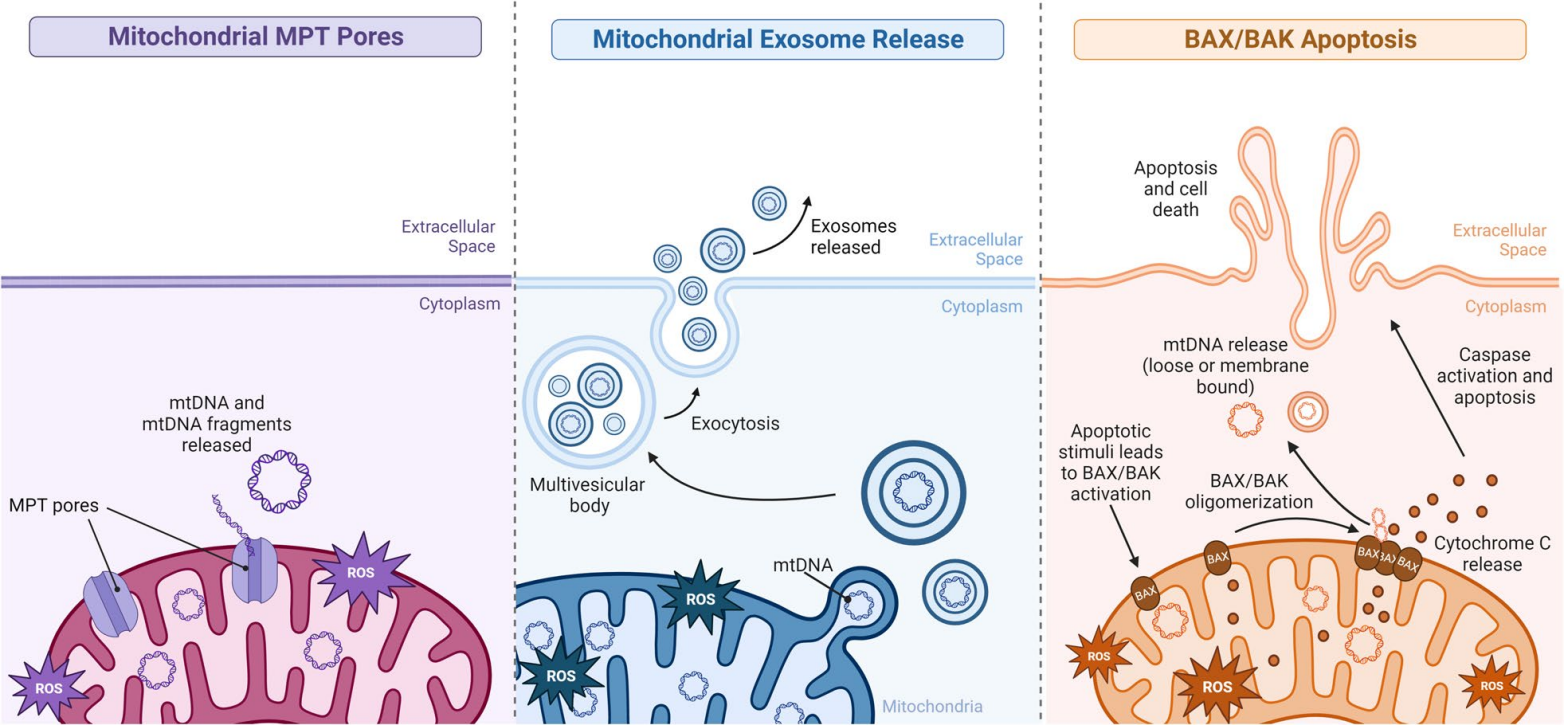
Mechanisms of mtDNA release. Stressed, dysfunctional, and/or damaged mitochondria may release mtDNA in whole or in fragments as a stress response via MPT pores, mitochondrial exosome release, and/or mitochondrial membrane permeabilization in BAX/BAK apoptosis. These mechanisms may cause a decrease in intracellular mtDNA and an accumulation of extracellular mtDNA which can act as a DAMP and trigger immune responses. This figure was created with BioRender.com

Reactive oxygen species (ROS), a byproduct of normal cellular function including the mitochondria’s electron transport chain, are normally kept in low concentrations under normal cellular conditions. However, if they are allowed to accumulate, these toxic molecules can precipitate oxidative damage to mitochondrial DNA, proteins, and lipids as well as increase mitochondrial dysfunction [14]. Increased levels of brain oxidative stress have been documented in AD [15, 16]. It is thought that increased levels of ROS are a main driver of mtDNA release from the cell [17–21]. Within the mitochondrial membranes, there is a non-specific pore formed by the association of several membrane proteins referred to as the mitochondrial permeability transition (MPT) pore [17]. The opening of this pore has been shown to allow the release of fragments of mtDNA under normal physiological conditions, but under pathological conditions, both the probability of pore opening as well as the amount of mtDNA released from the mitochondria increases significantly [17]. MtDNA has also been shown to be found in extracellular vesicles released from the cell, and there is some evidence that this process increases under pathological conditions, particularly in neurological disorders [18, 19]. As mitochondrial damage and dysfunction increase during states of oxidative stress, mtDNA is also shown to be released upon apoptosis via BAX/BAK signaling and mitochondrial membrane permeabilization [20, 21]. These are just a few of the ways that mtDNA has been shown to escape the mitochondria and the cell into the extracellular space (Fig. 1).

## Immunological effects

Brain inflammation has been well established as a signature of neurodegenerative diseases, particularly in AD [22, 23]. This is thought to be caused in part by the release of mtDNA and its function as a damage-associated molecular pattern (DAMP), which are known to trigger immune responses. Cell-free mtDNA is thought to function as a DAMP given its theorized endosymbiotic origins and similarity to bacterial DNA [24]. These mtDNA DAMPs have been shown to have a multitude of immunological effects. MtDNA has been shown to be related to an increase in the concentration of proinflammatory cytokines, particularly TNF-α and IL-6, which have been found to be elevated in aged individuals and those with dementia [12, 25, 26]. Fragments of mtDNA and oxidized mtDNA are known to lead to inflammasome activation, furthering the inflammatory response [27, 28]. Studies have also shown that mtDNA DAMPs can lead to neutrophil activation and recruitment as well as an increase in other innate immune responses [24, 29].

## Future directions

While there seems to be strong evidence for mtDNA functioning as a stress signal to activate the immune system in AD, there are still several questions remaining before we will be able to see the full picture. It is still unclear exactly what these observed alterations in mtDNA copy number indicate about the health of these tissues and what is going on at a cellular level. Alterations in mtDNA copy number may be due, at least in part, to variability in cell type proportions as an immune response particularly in complex tissues such as the brain and the blood. It remains unclear in the literature on whether mtDNA levels measured are altered due to immune responses or if immune responses are altered due to the presence and release of mtDNA extracellularly as proposed here. We believe the mechanism is likely some combination of both, causing a complicated cycle of mitochondrial stress and neuroinflammation. Future studies focused on identifying changes in mtDNA copy number in single cell types or the inclusion of data on cell type proportions may be particularly beneficial to furthering our understanding.

A secondary limitation to mtDNA copy number’s use as a biomarker is due to the inherent sources of mtDNA variability— across human population groups, over the life course, and throughout the body. Greater knowledge of how alterations in mtDNA concentrations vary across the cognitive spectrum (from unaffected, to MCI, to AD) as well as in various biological fluids/tissues is needed before it can be used as a biomarker. There is discussion in the literature over many of these points, due to variable reproducibility of results, small sample sizes, and a dearth of data across racial and ethnic groups. Previous studies will need to be confirmed to get a clearer picture of how mtDNA alterations may vary in different populations and future studies will be needed to further elucidate how these release mechanisms work and how exactly they are working within the context of AD. Lastly, further studies are needed into other possible effects of the extracellular mtDNA, both in the brain and circulating throughout the body, especially given that these molecules can be transported in exosomes and potentially taken up by targeted cells.

## Data Availability

Not applicable.

## Abbreviations

AD: Alzheimer’s disease
CFmtDNA: Cell-free mitochondrial DNA
CSF: Cerebrospinal fluid
DAMP: Damage associated molecular pattern
MPT pore: Mitochondrial permeability transition pore
mtDNA: Mitochondrial DNA
ROS: Reactive oxygen species
